# Mr. E. Brande, on a Poisonous Species of Agaric

**Published:** 1800-01

**Authors:** Everard Brande

**Affiliations:** No. 10, Arlington-street


### *To the Editors of the Medical and Phyſical Journal.*

GENTLEMEN,

IF the following account of the deleterious effects of a very common ſpecies of agaric, not hitherto generally ſuſpected to be poiſonous, appears to you likely to prove uſeful or intereſting to the public, you will oblige me by its inſertion; ſhould its length be any obſtacle to this, I beg you will omit whatever you may think ſuperfluous. I remain,

GENTLEMEN,

Your's, moſt obediently,

EVERARD BRANDE.

No. 10, *Arlington-ſtreet*,

*Nov. 16th*, 1799.

J. S. gathered early in the morning of the third of October, in the Green Park, what he ſuppoſed to be ſmall muſhrooms; theſe he ſtewed with the common additions in a tinned iron ſaucepan.* The whole did not exceed a tea ſaucerful, which he and four of his children ate the firſt thing, about eight o'clock in the morning, as they frequently had done without any bad conſequence; they afterwards took their uſual breakfaſt of tea, &c. which was finiſhed about nine, when Edward, one of the children, (eight years old,) who had eaten a large proportion of the muſhrooms, as they thought them, was attacked with fits of immoderate laughter, nor could the threats of his father or mother reſtrain him. To this ſucceeded vertigo, and a great degree of ſtupor, from which he was rouſed by being called or ſhaken, but immediately relapſed. The pupils of his eyes were, at times, dilated to nearly the circumference of the cornea, and ſcarcely contracted at the approach of a ſtrong light; his breathing was quick, his pulſe very variable, at times imperceptible, at others too frequent and ſmall to be counted; latterly, very languid; his feet were cold, livid, and contracted; he ſometimes preſſed his hands on different parts of his abdomen, as if in pain, but when rouſed and interrogated as to it, he anſwered indifferently, yes, or no, as he did to every other queſtion, evidently without any relation to what was aſked. About the ſame time the father, aged forty, was attacked with vertigo, and complained that every thing appeared black, then wholly diſappeared;

* This accuracy may ſeem trivial, but I have met with people who ſuppoſed the following ſymptoms might have ariſen from the uſe of a copper veſſel.

difappeared; to this fucceeded lofs of voluntary motion and ftupor; his pupils were dilated, his pulfe flow, full, and foft; breathing not affected; in about ten minutes he gradually recovered, but complained of univerfal numbnefs and coldnefs, with great dejection, and a firm perfuafion that he was dying; in a few minutes he relapfed, but recovered as before, and had feveral fimilar fits during three or four hours, each fucceeding one lefs violent and with longer intermiffions than the former.

Harriet, twelve years old, who had eaten but a very fmall quantity, was attacked alfo at the fame time with flight vertigo.

At nine o'clock I firft faw them, and ordered a folution of ten grains of tartar emetic, in four ounces of water, to be immediately given to each in proportioned dofes. It foon had the defired effect on the father and on Harriet, both of whom felt themfelves much relieved by its operation. As foon as the ftomach of the former could bear it, I ordered him an ounce of caftor oil, and half an hour afterwards, vinegar and water, of each two ounces. He took three fuch dofes, at intervals of half an hour, when he had a ftool, and voided large quantities of urine, and although not perfectly recovered, did not appear to require any thing more.

To Harriet, who had had two or three attacks of flight vertigo, with fome languor, I gave, (after the operation of the emetic,) on the fuggeftion of my friend, Dr. Burges, who happened to be prefent, thirty drops of fal volatile, in a table fpoonful of water. This relieved her exceedingly, and by repeating the dofe twice in the courfe of an hour, fhe was perfectly cured.

From the difficulty with which Edward was made to fwallow any thing, and from the large quantity required, it was eleven o'clock before he had taken enough of the emetic folution to excite vomiting; by this time the poifon had produced fo powerful an effect upon his fyftem, that he did not appear in the leaft relieved by it. I now ordered him a ftimulating injection, applied a blifter to his neck, and by degrees made him fwallow fome fmall quantities of fal volatile, diluted with no more water than was abfolutely neceffary; his feet were frequently rubbed with and wrapped up in warm flannels; in half an hour the injection was repeated; this foon procured two ftools, when he was fenfibly relieved, knew the voice of his father and mother, and complained of coldnefs and infenfibility about his ftomach. His whole abdomen was well rubbed before a fire with fome camphorated ftrong volatile liniment, which, at his own requeft, was repeated two or three times; he continued alfo to take the fal volatile, and fome caftor oil. By four o'clock every every violent symptom had left him, drowsiness and occasional giddiness only remaining, both of which, with some head-ach, continued during the following day.

Charlotte, a delicate little girl, ten years old, naturally of a most mild and tractable disposition, who also had eaten a large proportion, was suddenly attacked in the presence of Dr. Burges and myself, about half after ten, with vertigo and loss of voluntary motion; her pupils were very much dilated, and sight greatly impaired; these symptoms soon gave place to a degree of delirium, in which she refused to take any thing, forcibly striking whatever was offered to her. A blister was applied to her neck; and having given her a strong dose of the emetic solution, immediately on the first attack, which, though late, operated violently, she became composed as the sickness went off; and after taking a few doses of the sal volatile, was perfectly well, and wholly unconscious of any thing that had passed since the commencement of the symptoms; her pulse, which hitherto had not been much affected, was now irregular, and continued so, though in a less degree, during the whole of the day.

Martha, aged eighteen, who had eaten a small proportion, was attacked about eleven o'clock, with symptoms exactly the same as those of Harriet. She was treated in the same manner with similar success.

From the evident utility of determining the species to which these agarici belonged, I desired the man who had gathered and partaken of them, to bring me some of the same; and on inquiry found he had for several years been in the habit of gathering, in the same place, what he was confident were the same sort. Part of those which he brought me, I sent to Dr. Williams, Botany Professor of Oxford, to whom I had related the cases. In a note which he had the kindness to send me, he says, "Having since passed a short time, in company with Mr. "Sowerby,* he has compared them with the Fungi and plates "in his Museum. Mr. S. has no doubt respecting the spe-"cies; it appears to be a variety of the Agaricus glutinosus of "Curtis, (Flora Londinensis,) the same with Dr. Withering's "Agaricus semiglobatus, yet no notice is taken either by Curtis "or Withering of its deleterious quality. This may seem sin-"gular, as its effects were so strongly marked, unless any "mistake has been made by the person who collected the "specimens."

I have also examined some of the same parcel with Mr. Wheeler,

* Author of Coloured Figures of English Fungi.

Wheeler, Demonstrator of Botany of the Apothecary's Company, whose testimony concurring with the above, leaves no room to doubt their authority.

As some of your readers may not readily have an opportunity of referring to either of the authors already mentioned, I shall add Curtis's description of the species Agaricus glutinosus.

"Stalks generally single, sometimes clustered, from two to "four inches in height, the thickness of a goose quill, thread "shaped, whitish, almost solid; the tube being very small, "glutinous; ring, a little below the cap, scarce perceptible.

"Cap, from one to two inches in breadth, of a brown co-"lour; in the full grown ones hemispherical, always convex, "and more or less glutinous; wet with rain, it becomes browner "and transparent, so that it sometimes appears striated.

"Gills numerous, single, of a brownish purple colour, "clouded; whole ones about twenty, horizontal, three shorter "ones placed betwixt them; they throw out a powder of a "brownish purple colour."

With respect to the use of it, he only says, "There is no-"thing acrimonious or disagreeable in its taste, yet its appear-"ance will not recommend it to the lovers of mushrooms."

The variety, however, in question, (which is almost constantly to be met with on pasture land during autumn,) differs from this description chiefly in being of a conical form, as will be perfectly well seen in No. 19, of Mr. Sowerby's English Fungi, to be published on the first of January next; for the useful purpose of shewing which Mr. S. has expressly added figures 1, 2, and 3, of Table 248.

